# General practitioner (family physician) workforce in Australia: comparing geographic data from surveys, a mailing list and medicare

**DOI:** 10.1186/1472-6963-13-343

**Published:** 2013-09-03

**Authors:** Soumya Mazumdar, Paul Konings, Danielle Butler, Ian Stewart McRae

**Affiliations:** 1 APHCRI, Australian National University, Building 63, Cnr Mills and Eggleston Rds, Canberra, ACT 0200, Australia

**Keywords:** Primary health care, Geographical information systems (GIS), Spatial, Mailing lists, General practitioner (GP), Family physician, Data quality

## Abstract

**Background:**

Good quality spatial data on Family Physicians or General Practitioners (GPs) are key to accurately measuring geographic access to primary health care. The validity of computed associations between health outcomes and measures of GP access such as GP density is contingent on geographical data quality. This is especially true in rural and remote areas, where GPs are often small in number and geographically dispersed. However, there has been limited effort in assessing the quality of nationally comprehensive, geographically explicit, GP datasets in Australia or elsewhere.

Our objective is to assess the extent of association or agreement between different spatially explicit nationwide GP workforce datasets in Australia. This is important since disagreement would imply differential relationships with primary healthcare relevant outcomes with different datasets. We also seek to enumerate these associations across categories of rurality or remoteness.

**Method:**

We compute correlations of GP headcounts and workload contributions between four different datasets at two different geographical scales, across varying levels of rurality and remoteness.

**Results:**

The datasets are in general agreement with each other at two different scales. Small numbers of absolute headcounts, with relatively larger fractions of locum GPs in rural areas cause unstable statistical estimates and divergences between datasets.

**Conclusion:**

In the Australian context, many of the available geographic GP workforce datasets may be used for evaluating valid associations with health outcomes. However, caution must be exercised in interpreting associations between GP headcounts or workloads and outcomes in rural and remote areas. The methods used in these analyses may be replicated in other locales with multiple GP or physician datasets.

## Background

### Introduction

An equitably distributed primary healthcare workforce is key to an efficient healthcare system. Family Physicians or General Practitioners (GPs) form a vital component of this workforce. Inequities in the geographical distribution of GPs are associated with poorer health outcomes [1-3]. In Australia, where a large sparsely populated hinterland and remote communities create challenges for GP access [4] a small but growing literature is underscoring the importance of geographic access to GPs [5-7].

The quality of spatial GP data is integral to adequately examining geographic access to GPs. The aim of the analyses presented here is to explore the issue of spatial GP data quality by comparing various geographically explicit GP datasets in Australia with different conceptualizations of the workforce metric (headcounts and workload aware statistics). Further, in order to understand the effect rurality has on data quality we implement our analyses across different degrees of rurality. The following discussion outlines the relevant context to this analysis. We first describe the issues salient to spatial GP data quality. We then discuss geographical GP datasets in different jurisdictions followed by a short description of GP datasets in Australia. Finally, we discuss existing research on GP datasets in Australia and elsewhere.

### Geographic GP datasets: what are we measuring?

Two aspects of data quality are salient to GP accessibility studies. First is the geographic resolution or scale. If the available GP data are aggregated to coarse scales, for example the state level, then locally relevant analyses cannot be performed. Second, is the conceptualization of the workforce metric. While it is common to use GP headcounts or mere presence of a GP as a metric of GP access, there is evidence that this may produce misleading results [1]. In the Australian context, it is known that while the average GP work more hours per week with increasing rurality [8], there are also substantial numbers of GPs who provide short term locum services (henceforward called locum GPs) in rural Australia whose inclusion or exclusion from simple headcounts may skew workforce analyses [9]. Further, many GPs work in more than one location, and if these locations are in different geographic areas they can be counted in one or both of these areas providing potentially misleading information. In addition female GPs in Australia are more likely to work part time [8]. Recent research supports the use of alternative workload aware metrics such as the number of Full Time Equivalent (FTE) physicians. For example, in the United States the number of FTE GPs have been shown to be more strongly associated with health outcomes than GP headcounts [1]. Full Time Equivalent and Full-time Workload Equivalent (FWE) are two workload aware workforce metrics commonly used in Australia. The FWE metric, unlike FTE, does not “cap” doctors providing more than a standard full-time level of services at an upper threshold, usually of 1. Thus a GP providing 20% more than a standard full time level of services will be 1.2 FWE but 1.0 FTE [10]. However, a number of different methods of calculating FWE/FTE exist. Thus, it is important to have access to datasets that are at a high geographic resolution with pertinent GP workload information. Ideally, in order to achieve the most accurate understanding of GP workforce availability, data is needed at the individual practice address(es) level along with the total number of hours worked, patients seen and services rendered^a^. Such detail is rarely available.

### Geographic GP and physician datasets in the USA, Canada and Europe

Many countries have multiple sources of GP or physician data, with varying degrees of overlap, strengths and weaknesses. In the United States, limited datasets on FTE physicians geocoded to postcodes can be obtained from US-Medicare^b^. Individual physician address information can be obtained from the American Medical Association (US-AMA)^c^. Physician Masterfile. These datasets have been used in multiple analyses of relationships with outcomes [1,10-12]. Occasionally, surveys are also used to assess the geographical distribution of physicians [13]. In Canada, the Canadian Institute for Health Information (CIHI) aggregates physician benefits information from provincial government into a comprehensive database called the National Physician Database. This database offers a wealth of information on physicians geocoded to postcode of main activity. The information can also be used to calculate FTEs. CIHI also maintains the Scotts Medical Database which can be used to obtain physician headcounts. As in the United States, these datasets have been used to study relationships with various outcomes [14,15], and the geographical distribution of physicians [16,17]. Note that while the US-AMA Masterfile and Scotts Medical Database are privately sourced^d^, the US-Medicare data and National Physician Database are organized by public bodies. Given, the diversity of datasets, and the possibility of overlapping uses of these datasets, it is important that the degree of agreement or disagreement between them be understood. However, there has been limited effort in this direction either in the United States or Canada. High quality data on GP locations are available in the United Kingdom which have been used in a number of analyses [18,19]. GP address data are available from different sources in Ireland [7] and have been used to study issues of geographic access.

### Geographic GP and physician datasets in Australia in the context of Australia’s healthcare system

Similar to Canada and the United States, multiple sources of physician and GP data sources exist in Australia. However, unlike their North American counterparts data custodians in Australia operate a relatively restrictive data access regime and some data custodians do not release data at small geographies either to researchers or the public (see discussion). Also, unlike the CIHI in Canada, no Australian body functions as a centralized aggregator of physician data. These complications result in a greater multiplicity of datasets in the context of Australia’s health system.

The backbone of Australia’s healthcare system is Medicare. Medicare is tax-payer funded and offers universal insurance for private medical services. Almost all GP services in Australia are privately provided under a fee-for-service scheme with a rebate provided by Medicare at a level set by the Medicare Benefits Schedule (MBS) [20]. GPs may charge at the level of this rebate with no payment at point of service (approximately 80% of services in 2012) or may charge at a higher level with the patient paying the gap. This information on services provided can be used to infer GP workload (see Methods). Updated information on GPs registered for the Medicare program (which is almost all GPs in Australia) are held by the body that administers Medicare, the Department of Human Services. The Department of Health and Ageing (DoHA) also holds this data, and in addition to the data on headcounts of GPs derives measures of full time equivalence. These data are not publicly released at small geographies [21]. They are occasionally released for research [9,22] and other reports [23] but were not made available for these analyses [21].

In the absence of small area data from the Medicare data custodians, GP workforce data can be a) obtained from GP workforce surveys, b) obtained at relatively coarse geographic scales from the data custodian, c) derived indirectly from datasets reflecting numbers of services provided by GPs that are released by the Medicare data custodians, and d) obtained from private sources that use both internet based and traditional data gathering tools to create mailing lists of GPs. In the Australian context, all of these data sources are salient to enumerating the GP workforce. These datasets are discussed in greater detail in the methods-data section.

### Research on geographic GP and physician datasets

Different studies on the geographic distribution of GPs and related health workforce use different datasets. For example, while some studies use data from the Australian census [4,24], others use data from surveys [25], or state or territory health workforce registries [5,26]. While survey data may provide workload and other hard to obtain information, they may be less complete than registry data. In contrast, data from registries or established mailing lists are likely to be more comprehensive but lack workload information. Recently a number of studies of geographical access have made use of mailing list data [6,27]. While some studies attempt to take GP workload into account [6,25], other studies do not [5]. A majority of these studies are localized to specific geographic areas making comparisons across datasets difficult.

Some researchers have attempted to describe GP data sources [28-31] in Australia. One Australian [31], and one American study [32] have attempted to quantify the quality of physician datasets. The American study compared US-AMA data from a single state with records from the state registry and found the US-AMA database to be almost 100% complete. The Australian study used expert local knowledge of all GPs in the Northern Tasmania DGP, to compile a master/authority/baseline list of 139 active GPs. They arrived at this number by starting with a larger list compiled from various datasets and then culled all inaccurate entries. The researchers then attached two quality scores, sensitivity and predictive value positive with each GP dataset. While this is a valid approach to ascertaining the accuracy of a dataset, it also requires names and addresses to be present in multiple databases, a difficult proposition in a restrictive data access environment. Moreover, researchers are often interested in the quality of a dataset insofar as it affects the outcome of their analyses.

### Aims and objectives

Health researchers across jurisdictions are interested in investigating the relationship of GP access and availability to various health outcomes [33,34]. While there are a number of approaches to quantifying GP availability, GP density in a geographical area is a commonly used metric [33,34]. In Australia GP densities by geography have been used as a metric of GP demand and supply [22]. A relevant research question in this context is whether the choice of one GP dataset over another affects the results of an analysis. If the same outcome were being studied, this would be equivalent to studying the level of agreement between the various datasets. The aim of the analysis presented in this paper thus, is to explore how the various GP datasets in Australia compare across different geographies. More specifically, we are interested in evaluating the correlation of GP headcounts and total FTE/FWE GPs at different geographic scales, and in observing how these correlations vary with rurality or remoteness. We also compare total headcounts and FTEs/FWEs from the various datasets across states and territories.

This is intended to be an exploratory analysis of GP datasets, and it is anticipated that the results of our analyses will assist health services researchers in Australia to make informed choices about GP datasets. These analyses can be easily extended to other jurisdictions that have multiple sources of physician data and to other data sets if and when they become available. While some of the conclusions of the study are clearly limited to the Australian context and particular data sets, the broad conclusions of the study relating to the relative interchangeability or otherwise of data sets from different public and private sources, and data sets using different measures of GP workforce, for analytic purposes may be of relevance in other jurisdictions. In addition, countries such as Canada, Australia and to a lesser extent the USA, which have a large rural hinterland, may face similar issues with geographic data from rural areas.

## Methods

### General description of relevant datasets

Two surveys, one reported by the Australian Institute of Health and Welfare (AIHW) and another undertaken by Primary Health Care Research and Information Service (PHC RIS) provide annual estimates of the GP workforce at small geographies. DoHA, through the Public Health Information Development Unit (PHIDU) at the University of Adelaide releases the number of services billed by GPs annually through Medicare at small geographies. This information can be used to derive approximate FWE measures. Although the formal Medicare definition of a FWE GP depends on the value of services provided under the MBS, this can be approximated by the number of services billed by an average GP. While billing patterns vary from cost patterns to the degree that GPs provide different service mixes, these effects are relatively small and disappear at higher levels of aggregation. The total number of FWE GPs in these geographies can therefore be estimated. Note that if a GP provides publicly funded/non private services in a public hospital, then these services are not charged to or registered by Medicare [35]. In rural and remote areas GPs are more likely to provide services in public hospitals. Indirectly derived FWEs from Medicare data in these areas may thus be depressed.

**Figure 1:**
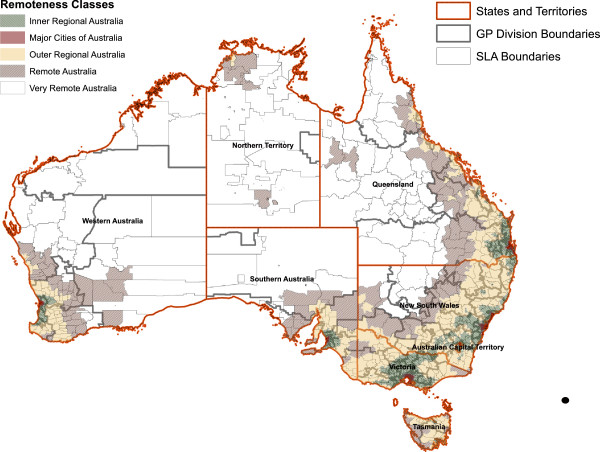
**Geographical Scales and Classifications: SLAs are nested in DGPs.** Median Population in SLAs is 4000 and 186,600 in DGPs.

None of these datasets provide information at scales finer than the Statistical Local Area (SLA). SLAs are geographies with populations varying from 0 to 130,000, with a mean population of 13,836 and a median population of 5,961. Compared to geographies from the USA, the wide variation in population sizes is comparable to the US Zip Code Tabulation Areas (ZCTAs) rather than the more homogenous US Census Tracts. The PHC RIS survey data are available at the scale of the Divisions of General Practice (DGP) also known as GP Network (GPN) in Australia^e^. The DGP or GPNs represent a geographical area of a functional and organizational network of GPs and GP practices. The data used in this analysis relates to 111 DGPs with a mean population of 199,120 and a median population of 186,660 (In the last 2 years DGPs have been replaced by larger bodies known as Medicare Locals; see discussion). DGPs encompass multiple SLAs and may encompass diverse rural and urban geographies. Figure 1 displays SLAs nested within DGP boundaries. For better geographic resolution, individual level address location information of GPs are available from mailing list management firms. One such firm is the Australasian Medical Publishing Company (AMPCo), whose “doctor lists” have been utilized in some studies [6,27], including a large scale longitudinal survey of the GP workforce in Australia [36]. Only full time or part time workload statuses of GPs are known in the AMPCo doctor list data, with no measure of actual hours worked. With the exception of the data from the AIHW survey data which is provided at SLA level and which are not publicly available, datasets are secondary and public, thus ethics clearances were not required for utilizing these data. An ethics clearance was obtained from the AIHW ethics committee (Reference number: EC 2010/2/23). The datasets analyzed in this study are described in Table 1. Detailed descriptions of these datasets, specific to our analyses are below.

**Table 1:** Sources of geographic GP datasets in Australia

**Dataset source**	**Geographic resolution**	**Coverage**	**Pros**	**Cons**	**Cost/Free**	**Source of information**	**Year of data used in analyses**
**Surveys:** Australian Health and Welfare (AIHW) Medical Workforce Survey	SLA (Statistical Local Area). SLA level data are available through special request only and at cost.	FTE GPs GP Headcounts	Extensive yearly survey of medical workforce. Sampling frame is all registered physicians and approximately 58,000 physicians answered the survey in 2007.	In SLA level data Estimates are missing/suppressed, either from non-response or privacy concerns from a small number of responses from large sections of rural Australia.	Cost	Survey (70% response rate in 2007)	2007
Primary Health Care Research and Information Service (PHCRIS) Annual Survey of Divisions (ASD)	DGP (Divisions of General Practice)	FWE GPs GP Headcounts	The survey had a 100% response rate from the 111 DGPs it was sent to in 2010.	DGPs occupy large geographies, thus requiring additional datasets to analyze within DGP variation.	Free	Survey (100% response rate from divisions in 2010). GP FWE Data is from DoHA	2010
**Mailing List:** Australasian Medical Publishing Company (AMPCo) “doctor Lists”	Individual points/ addresses/coordinates	Headcounts Full time or Part time	Excellent geographic resolution	Workloads of part time GPs are not known	Cost	Data acquisition method is not published.	2010
**Indirectly Derived:** DoHA/Social Atlas of Australia from Public Health Information Development Unit (PHIDU)	SLA	Number of services provided by GPs	Data provided by data custodian, thus valid and of good quality.	FWE has to be indirectly derived by diving the total number of services provided with the average number of services provided in a given year.	Free	Data obtained by PHIDU from DoHA	2009

### Specific description of datasets

#### Mailing list data

Preferred mailing addresses of GPs for the year 2010 were obtained from AMPCo. Mailing addresses do not necessarily correspond to GP practice addresses (see discussion for problems that may arise from this). AMPCo addresses were geocoded to derive individual latitude longitude coordinates. Of 23,261 addresses 23,170 were geocoded, 91 addresses that could not be geocoded were discarded. Of the 23,170 geocoded addresses, 23,118 could be attributed to SLAs. A visual inspection of 0.6% of addresses which could not be attributed to an SLA revealed they were distributed across all states of Australia. The raw data classified GPs into two categories; full time or part time. As a first approximation, we recoded all full time GPs as 1.0 and part time GPs as 0.5. We henceforth refer to this dataset as AMPCo doctor list.

#### Survey data

AIHW: We obtained SLA level Medical Workforce Survey data for the year 2007, of headcounts and FTE physicians held by AIHW. The overall survey response rate for 2007 was 69.9% [37]. Data at the SLA scale are not available in the published AIHW data and were obtained through special request. FTE is calculated by the AIHW as the sum of GP working hours in an SLA divided by full time work hours (45 hours a week). The AIHW survey sampling frame consists of a census of all registered physicians obtained from state and territory GP registration bodies^f^. Thus headcounts from this survey consist a complete enumeration. However, due to small cell count suppression for privacy concerns, a complete enumeration for all SLAs in Australia from the AIHW survey dataset was not available for this analysis. There were no records from one territory, the Northern Territory, because of a low response rate. Low response rates and unstable statistical estimates resulted in 274 SLAs (19%) showing missing values. We henceforth reference this data as AIHW survey.

PHC RIS: PHC RIS makes data from their Annual Survey of Divisions’ available on their website. This survey reports GP headcounts estimated from the survey in addition to GP FWE by DGP geography obtained from DoHA. One hundred and eleven DGPs were surveyed in 2010–2011 and the response rate was 100%.We obtained FWE GPs as of 30 June 2010, and GP headcounts from the 2010–2011 survey at the scale of the DGP [38]. We henceforth reference this as PHCRIS survey.

#### Indirectly derived FWE data

Social Health Atlas: Data on the total number of services billed by GPs at the scale of the SLA for the year 2009–10 was obtained from Social Atlas 2011^g^. Social Health Atlas data can be downloaded from the PHIDU website [39]. The number of services delivered in an SLA was divided by the total number of services billed by the average FWE GP in 2009 to obtain the approximate number of FWE GPs in an SLA. The number of services billed by the average FWE GP was obtained from the DoHA-Medicare data described below. We henceforth reference this data as indirectly derived FWE. FWE counts are not publicly available from Medicare at the SLA geography; hence we have indirectly estimated these numbers. Service numbers are obtained from Medicare by PHIDU through special request from the agency.

#### Baseline custodian data for comparison

We wish to compare the above datasets with an “authority” or “baseline” dataset. However, as discussed earlier such datasets are not readily available from custodians at fine geographic scales, but FTEs/FWEs can be compared against data available at a very coarse scale. GP workforce data aggregated by state and by ASGC (Australian Standard Geographic Classification) remoteness areas [40] for the year 2010 were extracted from the DoHA website [41]. ASGC remoteness areas are categorical metrics of rurality. Five categories of remoteness ranging from “major cities” to “very remote” exist. The categories reflect the distance to and the size of the nearest population center [42]. Figure 1 illustrates the geographical distribution of these areas. The DoHA statistics include all qualified GPs or other medical professionals that provided at least one un-referred attendance under Medicare [43] in 2010. Unreferred attendances include GP services that are provided by qualified medical practitioners who do not have a specific general practice qualification provided by the two professional colleges overseeing GP training in Australia. Since this definition of a GP is broad, it is expected that it will reflect more GPs by headcount than the other datasets. However, this definition will only minimally affect measures of FTE or FWE, because of the small overall number of unreferred services provided by the additional GPs. We henceforth reference this data as DoHA baseline data.

##### Analysis

The above datasets are available as excel tables with relevant geographical identifiers (such as SLA Identifier). They were attached to relevant Geographic Information Systems (GIS) geometries (ESRI, Environmental Systems Research Institute, shapefiles) downloaded from ABS. Since we wish to compare the datasets, it is necessary to scale the datasets to the same geography when there is a scale mismatch. Concordance tables were downloaded from the DoHA website to upscale the data at SLA scale to DGP scale. Attributes associated with SLA groups, a geography used in the PHIDU data, were given equal weights when decomposed to constituent SLAs. SLA groups are specific to the PHIDU data and were created to manage small populations in some SLAs. Headcounts and total FTE/FWEs were computed for all datasets, across states/territories, and ASGC remoteness categories. DoHA baseline data headcounts and FWEs were compared against headcount and FWE totals for the various datasets and percentage differences calculated. All analyses were accomplished with ESRI ArcGIS [44] and/or the R statistical software.

At each scale, Pearson’s correlation coefficients were calculated between headcounts and between FWE/FTE across datasets. Correlations between the AIHW survey and the other datasets at the SLA scale were calculated for only those SLAs for which the AIHW survey was not missing information. Thus the correlations exclude information from the Northern Territory. At each scale, correlations between the datasets were also calculated within ASGC remoteness categories. Data from the PHCRIS survey are at the DGP scale which encompass multiple remoteness categories and are excluded from the within-ASGC category correlation analysis. DGPs also occasionally cross state boundaries. To calculate FWE and headcount sums within states, DGPs need to nest in them. To achieve this, DGPs were decomposed to their component SLAs, and SLAs that crossed state boundaries were discarded.

Table 2 summarizes the correlation analyses that were implemented. Since the GP headcounts and FTE/FWEs are spatially autocorrelated, traditional metrics of confidence and p-values would be biased. One measure of spatial autocorrelation is Moran’s I, which ranges from −1 (indicating perfect negative correlation between neighbors), 0 (absence of correlation) to +1 (perfect correlation between neighbors) [45]. At the SLA scale Moran’s I is 0.33 (95% CI: 0.33, 0.34) for the AMPCo doctor list FTEs, 0.33 (95%CI: 0.32, 0.34) for AmpCo headcounts and 0.34 (95%CI: 0.34, 0.35) for indirectly derived FWEs. Efron’s bootstrap is one approach to estimating confidence intervals in data that are correlated, have outliers, and/or violate other distributional assumptions [46]. Thus, confidence intervals were created by bootstrapping to overcome spatial autcorrelation. One thousand replications were evaluated, each calculating the correlation from a randomly selected sample of 50% of the observations. This generates a histogram of correlation coefficients from which 95% confidence intervals were calculated.

**Table 2:** Each cell in this two by two table displays the datasets that are correlated against each other for a given scale-attribute combination, where the two scales are SLA and DGP, and the two attributes are headcounts and FTE/FWE

	**Attribute correlated**		
		**Headcounts**	**FWE/FTE**
**Geographic Scale of correlation**			
**SLA**		AMPCo doctor list,	AIHW survey,
		AIHW survey	AMPCo doctor list,
			Indirectly derived FWE
**DGP**		AMPco doctor list,	AIHW survey,
		AIHW survey,	AMPco doctor list,
		PHCRIS survey	Indirectly derived FWE
			PHCIRS survey

## Results

### Comparing GP headcounts by rurality and states

Compared to baseline data from DoHA, the various datasets have smaller headcounts in all regional categories. This is expected given the wider definitions of GPs used by DoHA based on the provision of unreferred services rather than the qualifications or professional role of the doctor. The AMPCo doctor list data uniformly overestimates FTEs over all categories of rurality, except in very remote areas which suggests that either not all part time GPs have their status reflected in the APMCo data, or our assumption of half time as 0.5 of full time is overstated, or quite probably both**.** The PHCRIS FWE numbers are almost the same as the DoHA numbers which is expected as they come from the same source. The FTE measures from the AIHW survey and the indirectly derived FWE data show a smaller number of FTE/FWE GPs in outer regional, remote and very remote areas compared to the DoHA published totals. The number of FTE GPs is same as the total number of GPs in both the AMPCo and AIHW data in very remote areas, implying that all represented GPs in these datasets in very remote areas report working full time. Derived estimates of FWE GPs from PHIDU show relatively small deviations from the DoHA baseline FWEs. The deviation increases with increasing rurality. Headcounts and FTE/FWE sums by states/territories, are provided in the Additional file 1: Table S1. The AMPCo doctor list and AIHW survey data show similar patterns as in Table 3, with smaller headcounts than the DoHA baseline data. The indirectly derived FWE shows the largest deviation from the DoHA baseline FWEs in the relatively rural parts of the Northern Territory.

**Table 3:** Headcount/FTE/FWE totals at different scales with percent deviations from DoHA baseline figures

	**Overall**		**Metro**		**Inner regional**		**Outer regional**		**Remote**		**Very remote**	
	**Headcount**	**FTE/FWE**	**Headcount**	**FTE/FWE**	**Headcount**	**FTE/FWE**	**Headcount**	**FTE/FWE**	**Headcount**	**FTE/FWE**	**Headcount**	**FTE/FWE**
AMPCo doctor list (FTE)												
*Summed total*	23,118	21,518	17,245	15,959	3,984	3,752	1,633	1,558	205	199	51	51
*% Difference from DoHA*	−13.13	30.55	−5.14	36.69	−22.41	17.36	−30.77	16.88	−60.95	1.53	−87.68	−37.65
AIHW survey (Overall from registers, FWE from survey)												
*Summed total*	21,817	18,623	16,538	13,813	3,711	3,306	1,321	1,259	192	191	55	55
*% Difference from DoHA*	−18.02	12.99	−9.03	18.31	−27.73	3.41	−44.00	−5.57	−63.42	−2.55	−86.71	−31.62
Indirectly derived FWE												
*Summed total*		19,688		14,593		3,426		1,443		162		63
*% Difference from DoHA*		−0.21		2.42		−6.56		−5.36		−22.10		−21.67
PHCRIS (Headcount from survey, FWE from DoHA)												
*Summed total*	24,688	20,049										
*% Difference from DoHA*	−7.23	−0.02										
DoHA	26,613	16,482 FTE	18,180	11,675 FTE	5,135	3,197 FTE	2,359	1,333 FTE	525	196 FTE	414	81 FTE
		19,729 FWE		14,248 FWE		3,667 FWE		1,525 FWE		208 FWE		81 FWE

### Correlations between counts of GPs

In general, there is excellent association between the various datasets, showing that while the levels of the estimates may vary the overall patterns between DGPs or between SLAs are similar. When segmented by remoteness, small numbers in remote and very remote areas mean that lower correlations are found, some of which are not significant. Generally, both the headcount and FTE/FWE attributes show strong significant correlations (Tables 4, 5 and 6). The correlations are preserved across scales. Since local variations tend to smooth out at coarser scales, correlations at the DGP scale are generally larger than at the SLA scale. However, there is greater variation in correlations when the data are segmented by rurality/remoteness. The strongest correlations are between the AIHW survey data and the AMPCo doctor list data. This is true for both the headcounts and the FTE measures. The patterns of overall correlation observed across the datasets are generally driven by the correlations in urban and inner regional areas that contribute the greatest degree of statistical power and most observations to the overall estimate. For example, at the scale of the SLA, there is an overall correlation of 0.88 (0.85-0.91) between AMPCo doctor list and AIHW survey FTEs, with coefficients of 0.85 (95% CI: 0.81-0.89) in metro, 0.87 (0.78-0.94) in inner regional and low of 0.52 (−0.42-0.91) in very remote areas. Correlations with the indirectly derived FWEs are somewhat weaker.

**Table 4:** AIHW survey, AMPCo doctor list and indirectly derived FTE/FWE are correlated at the DGP scale

				***AMPCo doctor list FTE***				
*AMPCo doctor list and AIHW survey*	***AIHW survey FTE***		*Metro*	*Inner Regional*	*Outer Regional*	*Remote*	*Very Remote*	*Overall*
		*Metro*	0.97(0.95,0.99)					
		*Inner Regional*		0.93(0.81,0.99)				
		*Outer Regional*			0.95(0.79,0.98)			
		*Remote*				0.83(0.55,0.99)		
		*Very Remote*					0.60(−0.89,1.00)	
		*Overall*						0.98 (0.96,0.99)
*Indirectly derived FWE and AIHW survey*	***AIHW survey FTE***			***Indirectly derived FWE***				
			*Metro*	*Inner Regional*	*Outer Regional*	*Remote*	*Very Remote*	*Overall*
		*Metro*	0.87(0.81,0.93)					
		*Inner Regional*		0.88(0.77,0.97)				
		*Outer Regional*			0.91(0.64,0.97)			
		*Remote*				0.82(0.56,0.99)		
		*Very Remote*					0.78(0.26,1.00)	
		*Overall*						0.90 (0.86,0.94)
*AMPCo doctor list and Indirectly derived FWE*	***Indirectly derived FWE***			***AMPCo doctor list FTE***				
			*Metro*	*Inner Regional*	*Outer Regional*	*Remote*	*Very Remote*	*Overall*
		*Metro*	0.82(0.74,0.90)					
		*Inner Regional*		0.95(0.93,0.98)				
		*Outer Regional*			0.97(0.84,0.99)			
		*Remote*				0.97(0.80,1.00)		
		*Very Remote*					0.47(−0.80,0.98)	
		*Overall*						0.87(0.81,0.92)

Not shown in this table are overall FWE/FTE correlations between in the PHCRIS survey and AIHW 0.82 (0.74, 0.88), PHCRIS survey, AMPCo doctor list 0.81 (0.72-0.88), PHCRIS survey and indirectly derived FWE 0.87 (0.80, 0.92).

**Table 5:** AIHW survey, AMPCo doctor list and indirectly derived FTE/FWE are correlated at the SLA scale

				***AMPCo doctor list FTE***				
*AMPCo doctor list and AIHW workforce survey*	***AIHW survey FTE***		*Metro*	*Inner Regional*	*Outer Regional*	*Remote*	*Very Remote*	*Overall*
		*Metro*	0.85(0.81,0.89)					
		*Inner Regional*		0.87(0.78,0.94)				
		*Outer Regional*			0.74(0.64,0.82)			
		*Remote*				0.58(0.38,0.93)		
		*Very Remote*					0.52(−0.42,0.91)	
		*Overall*						0.88 (0.85,0.91)
*Indirectly and AIHW workforce survey*	***AIHW survey FTE***			***Indirectly derived FWE***				
			*Metro*	*Inner Regional*	*Outer Regional*	*Remote*	*Very Remote*	*Overall*
		*Metro*	0.83(0.79,0.88)					
		*Inner Regional*		0.79(0.70,0.89)				
		*Outer Regional*			0.74(0.64,0.82)			
		*Remote*				0.63(0.49,0.89)		
		*Very Remote*					0.38(−0.64,0.83)	
		*Overall*						0.84 (0.81,0.88)
*AMPCo doctor list and Indirectly derived FWE*	***Indirectly derived FWE***			***AMPCo doctor list FTE***				
			*Metro*	*Inner Regional*	*Outer Regional*	*Remote*	*Very Remote*	*Overall*
		*Metro*	0.74(0.69,0.78)					
		*Inner Regional*		0.86(0.80,0.93)				
		*Outer Regional*			0.86(0.77,0.92)			
		*Remote*				0.86(0.52,0.97)		
		*Very Remote*					0.35(−0.48,0.96)	
		*Overall*						0.77 (0.74,0.81)

**Table 6:** AMPCo doctor list and AIHW survey headcounts are correlated at DGP and SLA scale

				***AMPCo doctor list headcount***				
*AMPCo doctor list and AIHW survey at DGP scale*	***AIHW survey headcount***		*Metro*	*Inner Regional*	*Outer Regional*	*Remote*	*Very Remote*	*Overall*
		*Metro*	0.98(0.96,0.99)					
		*Inner Regional*		0.94(0.80,0.99)				
		*Outer Regional*			0.97(0.82,0.99)			
		*Remote*				0.81(0.52,0.99)		
		*Very Remote*					0.38(−0.90,1.00)	
		*Overall*						0.98 (0.95,0.99)
*AMPCo doctor list and AIHW survey at SLA scale*	***AIHW survey headcount***			***AMPCo doctor list headcount***				
			*Metro*	*Inner Regional*	*Outer Regional*	*Remote*	*Very Remote*	*Overall*
		*Metro*	0.85(0.81,0.90)					
		*Inner Regional*		0.89(0.79,0.95)				
		*Outer Regional*			0.74(0.61,0.83)			
		*Remote*				0.57(−0.16,0.76)		
		*Very Remote*					0.30(−0.72,0.89)	
		*Overall*						0.87 (0.84,0.90)

## Discussion

### Our findings in context

The United States, Canada and Australia face unique challenges to their physician and General Practitioner workforce [8,47]. An increasing number of physicians are women who seek an optimal work-life balance and may work part time [48]. Rural areas face existing and new challenges to the GP workforce. Unless GP datasets reflect these changes, analyses of relationships between access to GPs and health outcomes shall be biased. In addition different spatial datasets may not be in agreement with each other. Our research shows, for the first time, that in the Australian context major sources of GP workforce data are in general agreement with each other at two geographic scales. We show that mailing list data are comparable to workforce information derived from surveys or indirectly derived from datasets released by data custodians.

Our analyses also underscore the particular nature of the GP workforce in rural and remote Australia. This is the one case where the use of data from different time points may have reduced the measured correlations, as in SLAs with few doctors an increase or decrease of one doctor between years may be material and may influence the correlations. There are however other factors influencing the correlations in the more remote areas. Firstly, as the private mailing list and the AIHW FTE measures are based on location of principle practice each GP is counted only once even if they work in multiple areas, while the DoHA FTE measures reflects the total number of services from GPs working in the area (from the MBS data). In rural areas, more services are provided by locum GPs or contracted fly-in fly-out services [8,35]. Also, many rural GPs work full time, but only part time as GPs in the private system under Medicare, with much of the remainder of their time spent in public hospitals [8]. The AIHW and AMPCo data are thus unable to capture the large number of part time GPs in rural and remote areas.

Our indirectly derived FWE measures are based on numbers of services while the DoHA FWEs are derivations based on service dollar values, if rural GPs provide a wider range of services and longer consultations than urban GPs then in the rural areas we would expect the indirect measures (which are based on number of services) to be lower than the formal DoHA FWE measures. It is likely that the issues of data quality in rural Australia are applicable to the United States and Canada. However, as discussed earlier there has been minimal effort to compare the relative quality of geographic GP and physician datasets in any of these jurisdictions. Problems with geographic data from rural areas in the United States are well known and have been shown to bias analyses of relationships with health outcomes or causes losses in statistical power [49].

### Other sources of GP data in Australia

While our analyses were implemented on a specific set of data, it is important to note that GP data are also available from the Australian Bureau of Statistics (ABS) and certain internet directories. The publicly available version of data released by ABS aggregates GPs, junior doctors and other doctors in training (such as registrars and interns) headcounts at small geographies. These aggregates coded as “Generalist Medical Practitioners” by the Australian Standard Classification of Occupations (ASCO), code number-2311, overestimate overall GP numbers by as much as 50% and is not a comparable enumeration of the GP workforce [50]. Also, certain internet based databases of health related locations such as physician clinics and practices provide some limited GP information. However, these data sources are of questionable quality and are often incomplete. A further data source which will be relevant for future analysis will the Australian Health Professions Registration Authority (AHPRA) which holds data on all registered medical professionals and health professionals in a range other professions. The data is not analyzed here as it has only been available for 2012 and it reflects only headcounts at the level of principal private practice. These data are also publicly available from Health Workforce Australia, an Australian government agency at the Local Government Areas which are comprised of one or more SLAs. The National Health Directory Service also collates and publishes information about various aspects of the health services system and workforce.

### The difficulty of obtaining locally relevant quality spatial GP data in Australia

Our research underscores the need for authority data at adequate geographic resolutions. While national workforce registrars such as AHPRA are a source of baseline data, the ultimate data custodians of Australia’s universal healthcare system are DoHA and DHS. These agencies do not generally publicly release FTE GP data at scales smaller than that of the state which are of limited if any use at all in a geographic analysis [21], although very occasionally data at fine scales are released on specific request for some research [9,22] and reports [23]. Contrast this, for example, with the CIHI database in Canada and the resulting research [16,17], research with US-AMA data [10,11,51,52] in the United states or GP data in New Zealand [53,54].

Local health agencies often benefit from the use of geographical information systems based planning and delivery [51]. In July 2011, the Australian Government created “Medicare Locals” , a set of 61 geographically bounded networks of primary healthcare organizations created for better delivery and organization of primary healthcare at a *local scale*, [55]. However, geographical analyses cannot be done at a local scale if the data that are supposed to drive these analyses are at coarse scales such as state or postcode [21]. Better resolution data will help researchers and policy makers map community level geographic variations within Medicare Locals. Medicare Locals are comparable to Primary Care Trusts in the United Kingdom, which serve the function of organizing geographically localized care. However, a researcher interested in analyzing workforce information within Primary Care Trusts has access to high quality practice level data, geocoded to the individual latitude longitude in addition to a host of other information on the practices and GPs [18,19].

In the absence of suitable custodian data in Australia, researchers and policy makers will frequently choose to utilize privately available data sources. One such dataset that offers good geographic resolution are mailing list data from AMPCo. However, as with any mailing list data, there are a number of shortcomings that need to be addressed. The first shortcoming is the coarseness of workload information. While mailing list data in the United States (The US-AMA master file) does not incorporate any workload information whatsoever, full time or part time information is available from the AMPCo doctor list data. However, coding part time GPs as 0.5 time, overestimates FTE GPs relative to DoHA baseline data. This implies that a number of part time GPs in the AMPCo mailing list data work at most less than half time. A second shortcoming is that it is not known to what extent AMPCo mailing addresses reflect locations of GP practices and not GP residences or other addresses. In the US context this difference is known to be significant and may potentially bias an analysis [56]. Finally, GPs are located to a single address and no information on shared practice locations was available on our version of the dataset.

### Other limitations

At the time of writing this paper, census ABS (2006) geographies such as SLAs are being transitioned to new geographies (2011) such as SA2 (Statistical Area 2) under the Australian Statistical Geography Standard (ASGS) census geography scheme. While SA2s have an average of 10,000 people compared to 14,000 per SLA, the SA2s are specially designed census geographies while the SLAs were based on administrative area (Local Government Area) boundaries. Also, General Practice Networks have been transitioned to Medicare Locals. While these transitions reflect significant changes in the organization of census data and local health networks, they are unlikely to influence the statistical correlations reported in this paper. If anything we expect the correlations to be greater at the scale of the Medicare Local given their much larger size relative to the DGPs.

Our results show that the available GP datasets are highly correlated across scales. Thus if a researcher were correlating or regressing GP densities against health outcomes using any of the datasets discussed in this paper, doing so at SLA or DGP scales should produce similar results. However, there are two important caveats that must to be taken into consideration. When two GP datasets (headcounts or FTEs) show a high “global” correlation over Australia, it does not imply that these correlations shall be equally high across small local areas. Thus, for example, two datasets may display very high correlation in one part of the country, but low correlation in another part, even though the overall ‘global’ correlation may remain high. When a single summary statistic such as a correlation coefficient is reported for an entire nation, local geographical variations in the statistic are concealed. While we address this issue to some extent by calculating different coefficients over different remoteness/rurality categories, this approach may be supplemented in the future with approaches that conceptualize space as a continuous surface. In this approach, “error maps” are used to visualize regions where two datasets are least (or most) in agreement. For example, McLafferty et al. [56] create two maps of physician density from two different datasets, and then subtracts one map from the other. The resulting error heat map allows McLafferty et al. to generate hypotheses on the drivers of data disagreement. Another approach to unpacking these relationships requires the use of Geographical Analysis Techniques such as Geographically Weighted Regression (GWR) which is a topic for further research [57].

The second caveat to note is that there is considerable mobility amongst GPs that is not obvious from our analysis. As the AIHW data are from 2007 while the remainder are from 2009–2010, we expect a number of GPs in the AIHW data to have moved to different locales by the time the other datasets were created. In spite of this, high correlations are observed between the AIHW and other datasets. There are two reasons for this. First, GPs moving out of a locale are replaced to some extent by GPs moving in. Second, systematic geographic patterns of GP movements are less visible in large geographies such as the SLA than in smaller geographies. Thus the results of these analyses should not be translated to finer scales.

## Conclusion

We have compared various General Practitioner datasets in Australia. These datasets are well correlated at the Statistical Local Area or Divisions of General Practice scales. However, caution must be exercised in evaluating and interpreting associations in rural and remote areas. Similar analyses can and should be implemented in jurisdictions that have multiple sources of GP or physician data to appropriately inform health services researchers.

## Endnotes

^a^Some GPs practice at multiple addresses over a time period

^b^US Medicare serves individuals older than 64, and a small number of other disabilities and conditions in the general population. Not all physicians accept Medicare patients, thus not all physicians are represented.

^c^We call the American AMA as US-AMA to distinguish it from the Australian Medical Association

^d^While Scott’s Medical Database is privately sourced, it is bought by CIHI annually

^e^The Australian General Practitioner’s Network or AGPN

^f^Australia introduced a single national physician registration body in 2010

^g^2010–11 data was not available at the time of writing this paper

## Abbreviations

GP: General practitioner; FTE: Full time equivalent; FWE: Full workload equivalent; US-AMA: American medical association; CIHI: Canadian institute of health information; MBS: Medicare benefits schedule; DHS: Department of human services (Australia); DoHA: Department of health and ageing (Australia); PHCRIS: Primary health care research and information system; AIHW: Australian institute of health and welfare; PHIDU: Public health information development unit; SLA: Statistical local area; DGP: Divisions of general practice; AMPCo: Australasian medical publishing company; ABS: Australian bureau of statistics; ASCO: Australian standard classification of occupations; AGPN: Australian general practice network; ASGC: Australian standard geographic classification; AHPRA: Australian health practitioner regulation agency.

## Competing interests

The authors declare that they have no competing interests.

## Authors’ contributions

SM implemented the analyses and wrote the paper. DB, ISM and PK conceived the analysis, collected the data and critically reviewed the manuscript. All authors read and approved the final manuscript.

## Pre-publication history

The pre-publication history for this paper can be accessed here:

http://www.biomedcentral.com/1472-6963/13/343/prepub

## Supplementary Material

Additional file 1: Table S1 Table enumerating correlation analyses implemented and table enumerating GP workforce by states and territories. Click here for file
