# Supplementary material for: General practitioner (family physician) workforce in Australia: comparing geographic data from surveys, a mailing list and medicare
Source: BMC Health Serv Res. 2013 Sep 3;13:343. doi: 10.1186/1472-6963-13-343 (PMC3766700; doi:10.1186/1472-6963-13-343)
Supplement: Additional file 1: Table S1 — Table enumerating correlation analyses implemented and table enumerating GP workforce by states and territories. [file 1472-6963-13-343-S1.docx]

Additional file 1: Table S1: Headcount (FTE/FWE) by state and territory with percentages deviation from DoHA baseline figures are provided below the number estimates^X^

| **Dataset** |  | **AMPCo doctor list** | | **AIHW survey** | | **Indirectly derived FWE** | **PHCRIS survey** | | **DoHA baseline data** | | |
| --- | --- | --- | --- | --- | --- | --- | --- | --- | --- | --- | --- |
|  |  | Headcount | FTE | Headcount | FTE | FWE | Headcount | FWE | Headcount | FTE | FWE |
| **New South Wales** | Total | 7,615 | 7130 | 6,861 | 6,029.27 | 6513.1 | 7,272 | 6,514 | 8,386 | 5,511 | 6,893 |
|  | % Difference from DoHA | -9.19 | 29.37 | -18.19 | 9.4 | -5.51 | -13.28 | -5.50 |  |  |  |
| **Victoria** | Total | 5,720 | 5,314 | 6,012 | 5,106.51 | 4621.13 | 6,207 | 4,915 | 6,451 | 4,121 | 4,902 |
|  | % Difference from DoHA | -11.33 | 28.94 | -6.81 | 23.91 | -5.73 | -3.78 | 0.27 |  |  |  |
| **Queensland** | Total | 4,615 | 4,294 | 3,844 | 3,244.71 | 3699.22 | 4,555 | 4,066 | 5,566 | 3,373 | 3,993 |
|  | % Difference from DoHA | -17.09 | 27.31 | -30.94 | -3.8 | -7.36 | -18.16 | 1.83 |  |  |  |
| **South Australia** | Total | 1,853 | 1,718 | 1,875 | 1,612.30 | 1409 | 2,295 | 1,570 | 2,204 | 1,329 | 1,546 |
|  | % Difference from DoHA | -15.93 | 29.27 | -14.93 | 21.32 | -8.86 | 4.13 | 1.55 |  |  |  |
| **Western Australia** | Total | 2,152 | 1,997 | 2,251 | 1,832.09 | 1555.25 | 2,116 | 1,441 | 2,491 | 1,436 | 1,614 |
|  | % Difference from DoHA | -13.61 | 39.03 | -9.63 | 27.58 | -3.64 | -15.05 | -10.72 |  |  |  |
| **Tasmania** | Total | 600 | 551 | 653 | 524.69 | 403.21 | 594 | 430 | 704 | 378 | 418 |
|  | % Difference from DoHA | -14.77 | 45.63 | -7.24 | 38.81 | -3.54 | -15.63 | 2.87 |  |  |  |
| **Northern Territory** | Total | 190 | 176 | 0 | 0 | 99.54 | 217 | 134 | 413 | 121 | 126 |
|  | % Difference from DoHA | -54 | 45.45 | 0 | 0 | -21.00 | -47.76 | 6.35 |  |  |  |
| **Australian Capital Territory** | Total | 372 | 339 | 321 | 273.73 | 230.07 | 463 | 239 | 398 | 212 | 238 |
|  | % Difference from DoHA | -6.53 | 59.91 | -19.35 | 28.77 | -3.33 | 16.33 | 0.42 |  |  |  |

X: AIHW data are from 2007.The data collected by the PHCRIS survey is at geographies (DGPs), - a scale that encompasses multiple remoteness areas, and therefore cannot be categorized by remoteness. Percentage errors for the AIHW survey and APMCo doctor list FTEs are calculated against DoHA baseline FTEs, while DoHA baseline FWEs are used for the remaining two datasets.
